# Mismatch repair and treatment resistance in ovarian cancer

**DOI:** 10.1186/1471-2407-6-201

**Published:** 2006-07-31

**Authors:** Jozien Helleman, Iris L van Staveren, Winand NM Dinjens, Patricia F van Kuijk, Kirsten Ritstier, Patricia C Ewing, Maria EL van der Burg, Gerrit Stoter, Els MJJ Berns

**Affiliations:** 1 Department of Medical Oncology, Erasmus MC/Daniel den Hoed Cancer Center, Rotterdam, The Netherlands; 2 Department of Pathology, Erasmus MC/Daniel den Hoed Cancer Center, Rotterdam, The Netherlands; 3 Erasmus MC, Department of Medical Oncology, Josephine Nefkens Institute, Room Be424, P.O. Box 1738, 3000 DR, The Netherlands

## Abstract

**Background:**

The treatment of ovarian cancer is hindered by intrinsic or acquired resistance to platinum-based chemotherapy. The aim of this study is to determine the frequency of mismatch repair (MMR) inactivation in ovarian cancer and its association with resistance to platinum-based chemotherapy.

**Methods:**

We determined, microsatellite instability (MSI) as a marker for MMR inactivation (analysis of BAT25 and BAT26), MLH1 promoter methylation status (methylation specific PCR on bisulfite treated DNA) and mRNA expression of MLH1, MSH2, MSH3, MSH6 and PMS2 (quantitative RT-PCR) in 75 ovarian carcinomas and eight ovarian cancer cell lines

**Results:**

MSI was detected in three of the eight cell lines i.e. A2780 (no MLH1 mRNA expression due to promoter methylation), SKOV3 (no MLH1 mRNA expression) and 2774 (no altered expression of MMR genes). Overall, there was no association between cisplatin response and MMR status in these eight cell lines.

Seven of the 75 ovarian carcinomas showed MLH1 promoter methylation, however, none of these showed MSI. Forty-six of these patients received platinum-based chemotherapy (11 non-responders, 34 responders, one unknown response). The resistance seen in the eleven non-responders was not related to MSI and therefore also not to MMR inactivation.

**Conclusion:**

No MMR inactivation was detected in 75 ovarian carcinoma specimens and no association was seen between MMR inactivation and resistance in the ovarian cancer cell lines as well as the ovarian carcinomas. In the discussion, the results were compared to that of twenty similar studies in the literature including in total 1315 ovarian cancer patients. Although no association between response and MMR status was seen in the primary tumor the possible role of MMR inactivation in acquired resistance deserves further investigation.

## Background

Ovarian cancer is the leading cause of death from gynecological cancer in the Western world [1]. The treatment of ovarian adenocarcinoma has improved over the last 20 years owing to the combined treatment of cytoreductive surgery and chemotherapy [2]. Although the response of the primary tumor to taxane and platinum-based chemotherapy is high, about 20% of patients never achieve a clinical response and the majority of the patients will relapse and eventually die of drug-resistant disease [3].

If it would be possible to predict primary platinum resistance, patients might be spared an ineffective but toxic platinum-containing therapy and might benefit from an early therapy with different drugs. Recently, several molecular profiling studies, including our study, have revealed gene sets that can predict response to platinum-based chemotherapy in ovarian cancer [4-6]. We discovered a nine-gene set which predicts response with a sensitivity of 89% and a specificity of 59% [5]. One of these nine genes was proliferating cell nuclear antigen (PCNA). PCNA is a DNA sliding clamp that interacts with several proteins involved in cell cycle control, DNA methylation, DNA replication and DNA repair including mismatch repair [7]. In this study, we have focused on DNA mismatch repair and its role in platinum-based chemotherapy resistance in ovarian cancer.

**Figure 1:**
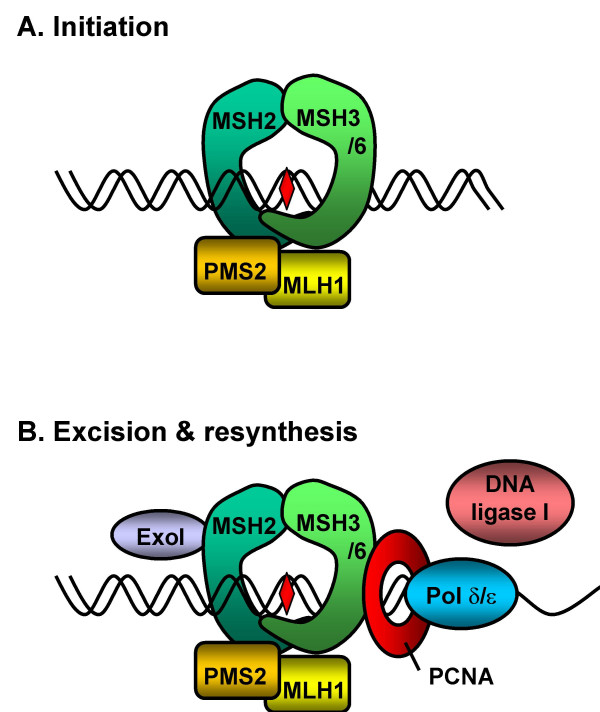
**The mismatch repair system (MMR)**. A. Based on figure 3 from Bellacosa et al [8]. Initiation of MMR by recognizing the DNA damage by the MutSα or β complex and recruiting the MutLα complex. B. Excision of the damaged strand and resynthesis in which exonuclease ExoI, proliferating cell nuclear antigen (PCNA), DNA polymerase δ or ε and DNA helicase I are suggested to play a role.

DNA mismatch repair (MMR) is divided into three steps: initiation, excision and resynthesis (Figure 1). Several proteins are involved in the initiation of MMR including the three MutS-homologs, MSH2, MSH3 and MSH6. The MutS homologs form a heterodimer that recognizes DNA damage; the MSH2 and MSH6 dimer (the hMutSα complex) recognizes base-base mismatches and single base loops whereas the MSH2 and MSH3 dimer (hMutSβ complex) recognizes insertion/deletion loops of more then one base. After the recognition of the DNA damage the binding of a heterodimer of the MutS-homologs MLH1 and PMS2 (the hMutLα complex) leads to the further initiation of MMR. Other known and still unknown proteins involved in the last two steps of MMR, the excision of the damaged strand and the resynthesis, are recruited subsequently. Proteins known to be involved are exonuclease ExoI, proliferating cell nuclear antigen (PCNA), DNA polymerase δ and perhaps ε and in addition based on its association with DNA polymerase δ and PCNA, DNA ligase I [8,9].

Inactivation of MMR leads to the occurrence of unrepaired deletions in mono- and dinucleotide repeats resulting in variable lengths of these repeats. This is called microsatellite instability (MSI) and MSI is therefore used as a marker for MMR deficiency. MSI can be caused by genetic or epigenetic inactivation of several genes involved in MMR. Mouse knockout models have demonstrated that MSH2^-/-^, MSH3^-/-^, MLH1^-/- ^and PMS2^-/- ^leads to a high frequency of MSI while MSH6^-/- ^and PMS1^-/- ^cause a low frequency (reviewed by Wei et al. [10]). However, in hereditary nonpolyposis colon cancer (HNPCC) families (which are known to have a high frequency of MSI) germline mutations in MSH2 and MLH1 are responsible for the MSI, while MSH6 and PMS2 are less frequently involved [9]. The lesser role for MSH6, PMS2, PMS1 and especially MSH3 inactivation in MSI seen in HNPCC patients could be due to functional redundancy of these genes.

Interestingly, a number of *in vitro *studies have suggested a relationship between MMR deficiency and platinum-drug resistance. Several resistant sublines of ovarian and melanoma cell lines generated by cisplatin selection, appeared to be MMR deficient [11-15]. In addition, a colon (HCT116) and an endometrioid cell line (HEC59), deficient for MLH1 and MSH2 respectively, were 2.1 and 1.8 fold resistant to cisplatin compared to cell lines complemented with chromosome 3, containing MLH1, or chromosome 2, containing MSH2 [15-17]. These *in vitro *studies suggest that inactivation of proteins involved in the initiation of MMR might cause cisplatin resistance. It is thought that the DNA damage caused by platin-drugs is recognized by MMR. The cell will then undergo several unsuccessful repair cycles, finally resulting in the induction of apoptosis. When MMR is inactive the DNA damage caused by platin-drugs will not be picked up and will therefore not result in apoptosis rendering the cells resistant to platin-drugs.

Several studies have determined the frequency of MMR inactivation in ovarian cancer using MSI as a marker [18-37]. However, there was a wide range observed (0%–39%) and so far only a few studies have linked MMR inactivation to platinum-based chemotherapy resistance. Thus, there is still no general agreement about the frequency of MMR inactivation and its possible involvement in the platinum-based chemotherapy resistance seen in ovarian cancer patients.

The aim of this study is to determine the frequency of mismatch repair (MMR) inactivation in ovarian cancer and whether it is associated with platinum-based chemotherapy resistance. To this purpose we analyzed seventy-five ovarian carcinomas and eight ovarian cancer cell lines. In the discussion, the results were compared to that of twenty similar studies in the literature including in total 1315 ovarian cancer patients.

## Methods

### Cell culture

All cell lines were cultured in medium supplemented with 100 U/ml penicillin, 100 μg/ml streptomycin and 50 μg/ml gentamycin at 37°C in humidified air with 5% CO_2 _(except for SW48 which was cultured with 10% CO_2_). The human ovarian cancer cell lines SKOV6, HOC7, SKOV3, 2774, KB3.1 and CAOV3 were cultured in DMEM/HAMF12 medium with 10% fetal calf serum, A2780 in RPMI 1640 medium with 10% fetal calf serum and OVCAR3 in RPMI 1640 with 20% fetal calf serum and 0.01 mg/ml insulin. The human colon cancer cell lines SW480 and SW48, included as controls, were cultured in RPMI 1640 with 5% fetal calf serum and DMEM/HAMF12 with 10% fetal calf serum respectively. The ovarian cancer cell line A2780 has been cultured separately in two different research laboratories at our department. The isolated DNA and RNA from each culture were used for further analysis.

The MTT colorimetric assay, which measures the number of viable cells capable of reducing the tetrazolium compound (Sigma-Aldrich, Zwijndrecht, The Netherlands) to a blue formazan product, was used to quantitate the chemosensitivity of the ovarian cancer cell lines to cisplatin. The assay was performed as described previously by us [38].

### Patients

The study design was approved by the medical ethical committee of the Erasmus MC Rotterdam, the Netherlands (MEC 02.949). Tissue of 75 ovarian cancer patients and four normal stromal ovarian tissues collected at the Erasmus MC in Rotterdam were included in this study. The patient and tumor characteristics are listed in Table 1. Forty-six patients received platinum-based chemotherapy of whom 34 responded to treatment defined as complete response, partial response, stable disease or no relapse within 6 months after chemotherapy, whereas eleven patients had progressive disease or a relapse within 6 months after chemotherapy. In one patient the response was not known. The response rate of 74% (34/46) is comparable with the response rate of 80% seen in the clinic. A more detailed description of the response definitions has been previously described by us [5]. The median age at the time of surgery was 52 years (range 27–83).

### DNA isolation: microsatellite analysis and methylation specific PCR

**Figure 2:**
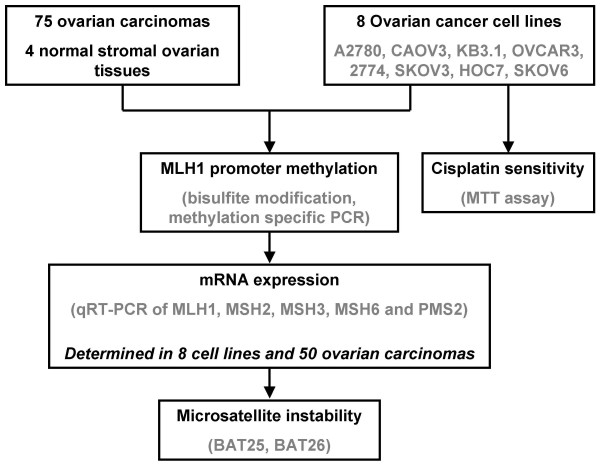
**Study design**. Flow chart for study design.

Microsatellite analysis and methylation specific PCR (MSP) were performed on DNA from eight ovarian cancer cell lines, 75 ovarian cancer specimens (part of a collection of ovarian tumor specimens described by us previously [39]) and the four normal stromal ovarian specimens (see study design in Figure 2).

Microsatellite analysis was standard performed in our laboratory as described by Westenend et al [40] using the two mononucleotide markers, BAT25 and BAT26. In addition, the 75 ovarian carcinomas were also analysed with the mononucleotide marker BAT40 (n = 42) or with the dinucleotide marker D2S123 (n = 40). So all ovarian carcinomas were analysed with three or four MSI markers. A PCR containing α-^32^PdATP, was performed on 100 ng DNA. PCR products were separated on a denaturing 6% polyacrylamide gel. After electrophoresis, gels were dried on blotting paper on a vacuum gel dryer and exposed to x-ray film. The films were evaluated by visual inspection.

The methylation specific PCR (MSP) was used to determine the promoter methylation of MLH1 after the DNAs were modified with sodium bisulfite using the Ez DNA methylation kit (Zymo research). We designed and optimized primers that are specific for methylated and unmethylated CpG islands within the MLH1 promoter (methylated: Forward 5'-CGAATTAATAGGAAGAGCGGATAGC-3', Reverse 5'-ACCTCAATACCTCGTACTCACG-3'; unmethylated: Forward 5'-TGAATTAATAGGAAGAGTGGATAGT-3', Reverse 5'-CCTCAATACCTCATACTCACA-3'). Both primers are located within a region important for a maximal transcription of MLH1 (including the binding site for the transcription factor CBF) [41,42], since methylation at this region is most likely to inhibit transcription of the gene. The PCR mixture contained 1× PCR buffer (as described by Herman et al [43]), dNTPs (each at 5 mM), primers (1 pmol/μl each per reaction), *Taq *polymerase (0.05 U/μl) and 100 ng modified DNA in a volume of 25 μl. Amplification was carried out for 35 cycles (30 sec 95°C, 30 sec 58°C for methylated and 55°C for unmethylated and 30 sec 72°C) followed by a final 4 minutes extension at 72°C. Controls without DNA were performed and in addition, the colon cancer cell lines SW48 with a methylated MLH1 promoter and SW480 with an unmethylated MLH1 promoter, were used as positive and negative control respectively.

### Quantitative RT-PCR

Quantitative RT-PCR analysis was used to measure the mRNA expression levels of MLH1, MSH2, MSH3, MSH6 and PMS2 in the eight ovarian cancer cell lines and 50 of the 75 ovarian cancer specimens of which RNA was available. Thirty-six of these 50 patients received platinum-based chemotherapy (7 non-responders, 28 responders and one patients with unknown response). The following 20× assay-on-demand primers and FAM-TAMRA labeled probe-mix from Applied Biosystems were used; for MLH1 (Hs00179866_m1), MSH2 (Hs00179887_m1), MSH3 (Hs00267239_m1), MSH6 (Hs00264721_m1) and PMS2 (Hs00241053_m1).

## Results

### Microsatellite analysis

Two sublines of the ovarian cancer cell line A2780 that have been cultured by two research groups in our department and the cell lines SKOV3 and 2774 showed a microsatellite instable (MSI) pattern for both mononucleotide markers BAT25 and BAT26. All other cell lines showed no aberrations. In addition, the 75 ovarian carcinoma tissues and the four normal stromal controls showed no aberrations for BAT25, BAT26 and BAT40 or D2S123, indicating that these are microsatellite stable (MSS).

### MLH1 promoter methylation

One of the two A2780 sublines showed complete methylation of the MLH1 promoter while the other showed a low level of methylation. The results for HOC7 and 2774 were not informative and the other five cell lines showed no methylation. A low level of MLH1 promoter methylation was also seen in six ovarian carcinoma specimens and in addition, one ovarian carcinoma specimen showed abundant methylation. Five ovarian carcinomas were not informative while the other 63 ovarian carcinomas showed no methylation.

### Quantitative RT-PCR: expression of MLH1, MSH2, MSH3, MSH6 and PMS2

**Figure 3:**
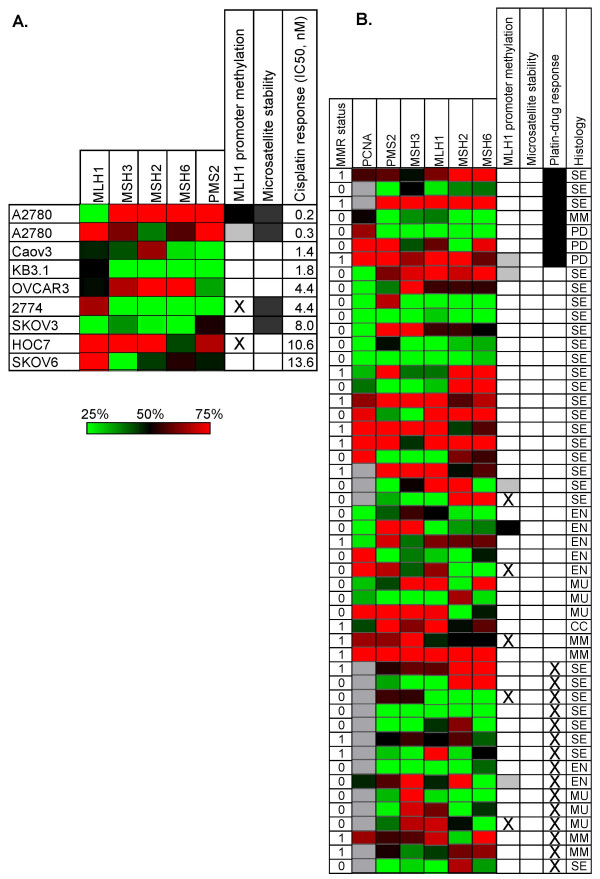
**The mRNA expression data for A. eight ovarian cancer cell lines, and B. 50 ovarian carcinomas**. The ovarian cancer cell lines and the ovarian carcinomas are ordered according to their cisplatin and platinum-based chemotherapy response. The MMR status deducted from the mRNA expression levels is given for the carcinomas (1: active, 0: inactive). The mRNA expression is shown in the heatmap (green color: low expression (25^th ^percentile calculated per gene), black: median expression, red color: high expression (75^th ^percentile calculated per gene), gray: no value). Depicted next to the heatmap is: the MLH1 promoter methylation status (black: complete or high level, gray: low level, white: no methylation, X: unknown), the microsatellite stability (black: instable, white: stable, X: unknown), the cisplatin response (Figure 3A; IC50 in nM) or platinum-based chemotherapy response (Figure 3B; black: non-responders, white: responders, X: unknown) and the histology (PD: poorly differentiated, SE: serous, CC: clear cell, MU: mucinous, EN: endometrioid, MM: mixed mullerian). Cell lines and carcinomas are ordered according to their cisplatin response or platinum-based chemotherapy response respectively.

The mRNA expression data for the cell lines is shown in Figure 3A. One of the two separately cultured MSI positive A2780 cell lines showed complete methylation of the MLH1 promoter and had no mRNA expression of MLH1. The other A2780 showed a low level of methylation but had the highest MLH1 expression levels compared to the other cell lines. Of the other two MSI positive cell lines, SKOV3 also showed no MLH1 expression while 2774 did express MLH1 mRNA.

RNA was available for 50 of the 75 ovarian carcinomas and the mRNA expression data for these carcinomas is shown in Figure 3B. Interestingly, the ovarian carcinoma with an abundant MLH1 promoter methylation had a low MLH1 mRNA expression compared to the other carcinomas. Thirty-six of the 50 patients received platinum-based chemotherapy (7 non-responders, 28 responders and one patient with unknown response). There was no significant association between the response to platinum-based chemotherapy and the expression of each of these genes separately (Mann-Whitney test, p > 0.6). Since inactivation of only one of these genes might be sufficient to cause MMR deficiency, we used the expression of all of the five genes to mark MMR as active or inactive. If at least one of the five genes had an expression in the lowest quartile we marked MMR as inactive. If none of the genes had an expression in the lowest quartile MMR was marked as active (Figure 3B). Next the Mann-Whitney test demonstrated that there was no significant relation between the deducted MMR status and response to platinum-based chemotherapy (p = 0.665).

## Discussion

In this study we aimed to address two questions; 1) what is the frequency of MMR inactivation in ovarian cancer, and 2) is it associated with platinum-based chemotherapy response.

First we analyzed eight ovarian cancer cell lines, i.e. SKOV6, HOC7, SKOV3, 2774, OVCAR3, KB3.1, CAOV3 and A2780. Microsatellite instability (MSI), which is a marker for MMR inactivation, was detected in three out of eight cell lines i.e. SKOV3, 2774 and A2780. This results in a frequency of MMR inactivation in ovarian cancer cell lines of 38%. The MSI in SKOV3 can be explained by the loss of MLH1 mRNA expression which, however, was not caused by promoter methylation. This is in agreement with the loss of MLH1 protein expression seen in SKOV3 described in a study of the 60 NCI cancer cell lines [44]. In concordance with our findings, 2774 was also described to be MSI [45]. One of the MSI positive A2780 sublines showed a strong methylation of the MLH1 promoter without MLH1 mRNA expression, while the other subline showed a low level of methylation and relative high mRNA expression. Strathdee et al. described that one MLH1 allele was methylated in A2780 [12] which is comparable with the methylation status we saw in A2780, moreover one of our A2780 sublines showed complete methylation. On the other hand, another study did not detect MSI in A2780 [11]. Interestingly, Aquilina and colleagues suggested there is a subpopulation of A2780 cells, estimated to be around one per 10^6 ^cells [46], which are MLH1 deficient and heterozygous for the p53phe172 mutation [46,47]. Since these cells have a growth advantage, prolonged culturing of the A2780 cell line can result in selection of this subpopulation. Thus over time, separately cultured A2780 can have varying percentages of cells belonging to this subpopulation which may explain the discrepancies in MMR status seen in the A2780 cell lines analyzed by us.

Next we studied the association between MMR inactivation and cisplatin resistance in these cell lines. MMR inactivation seen in SKOV3 and 2774 might result in the relative resistance to cisplatin compared to the other cell lines. On the other hand, A2780 which has clearly an inactive MMR, was most sensitive to cisplatin. Overall, there seems to be no association between the response to cisplatin and MMR status in these eight cell lines. This is similar to a study in the 60 NCI cell lines which also showed no association between response to cisplatin and MMR status based on the MLH1 and/or MSH2 protein expression [44].

Furthermore, we analyzed MMR status in 75 ovarian carcinomas to determine the frequency of MMR inactivation in ovarian cancer *in vivo*. Seven of the 75 ovarian carcinomas showed MLH1 promoter methylation. We confirmed whether the observed MLH1 promoter methylation results in the inactivation of the gene by determining the MLH1 mRNA expression with quantitative RT-PCR. The six tumors with low level MLH1 promoter methylation appeared to express MLH1 at mRNA levels similar to that of the unmethylated tumors. Thus a low level of methylation does not result in an altered expression of the MLH1 gene. In contrast, the abundant methylation seen in the remaining carcinoma was associated with the lowest MLH1 mRNA expression level of all 50 ovarian carcinomas tested. However, none of the ovarian carcinomas showed MSI for BAT25, BAT26 and for BAT40 or D2S123 which suggests a frequency of MMR inactivation of 0%. The low MLH1 mRNA expression seen in the abundant methylated carcinoma might be sufficient enough for a functional MMR which results in the observed absence of MSI.

Since ovarian cancer is a heterogeneous disease characterized by various histological types which may have different MSI frequencies, the number of specimens analyzed is very important in characterizing a feature that may be uncommon such as MSI. We therefore, made a summary of 20 studies totaling 1315 ovarian carcinomas, to compare the findings of these studies with our results (Table 2). The MSI frequencies determined in these studies ranged from 0% to 39%. Overall, MSI was detected in 165 of the 1315 ovarian carcinomas tested, suggesting an overall incidence of 13% [18-37].

Multiple differences between these studies could have caused the wide range in the MSI frequency (0–39%). One of these is the number and variety of microsatellite markers analyzed to determine the MSI. The NCI recommended five markers comprising the National Cancer Institute Consensus Panel (NCI-CP) for the detection of MSI, i.e. markers for the mononucleotide repeats BAT25 and BAT26 and the dinucleotide repeats D2S123, D5S346 and D17S250 [48]. Table 2 shows per study the number of MS markers used and specifies how many of these are part of the NCI-CP. Interestingly, the studies that used all NCI-CP markers to determine the MS status also showed a wide range in MSI frequency (8–39%) which is similar to the overall range (0–39%). Therefore, the various MS markers used cannot be the sole cause for the wide range. Moreover, Gras et al. suggest that the reliability of the mononucleotide markers BAT25 and BAT26 is so high that most MSI can be predicted by evaluating these two markers exclusively [27], confirming the less stringent role for the various markers used for the analysis.

Another difference between the studies is the distribution of the various histological types of the ovarian carcinoma tissues analyzed (Table 2). This difference in the distribution could be a cause for the wide range in the MSI frequency especially since it has been suggested that certain histological types have a higher frequency of MSI. To determine whether there is a relation between histology and MSI within these studies, we looked at the frequency of MSI per histological type for the 628 patients with known histology (Table 2). The summary of these studies suggests that the frequency of MSI is higher in the mucinous and endometrioid adenocarcinoma compared to clear cell and serous adenocarcinoma (the overall frequencies of MSI were 22%, 16%, 9% and 8%, respectively) (Table 2). We hypothesize that mucinous and endometrioid histology might be prone to a higher MSI frequency since sporadic endometrial carcinoma, which is closely related to endometrioid ovarian cancer, has a MSI frequency of 20–30% [49-51] and MSI is almost universal present in the colorectal tumors of the hereditary nonpolyposis colon cancer (HNPCC) syndrome which all have a mucinous histological type. Therefore, the different histology's of the ovarian carcinomas included in the several studies seems to be a plausible cause for the wide range in MSI frequency reported in these studies.

Next we addressed the second part of the aim of this study, is MMR inactivation associated with resistance to platinum-based chemotherapy in ovarian cancer. Forty-six of the 75 ovarian carcinomas we analyzed had been treated with platinum-based chemotherapy, eleven did not respond and 34 did. For one patient the response was not known. Methylation of the MLH1 promoter was detected in two of the eleven non-responders (18%) and four of the 34 responders (12%) and this was not significantly different (p = 0.664). Since we did not detect any MSI, the resistance seen in the eleven patients could not be associated with MSI and MMR inactivation.

The relation between MMR deficiency and platinum-drug resistance has been investigated in only a few *in vivo *studies. Similarly to our result, no MSI was detected by Mesquita et al. [18] who studied 34 ovarian carcinomas of which seven did not respond to cisplatin/paclitaxel therapy. So the resistance seen in these seven nonresponding patients was also not associated with MMR inactivation. In contrast, Samimi et al. [52] found an inverse relation between MLH1 protein expression and the response to platinum-based chemotherapy in 54 ovarian carcinomas. Again, the number of ovarian carcinomas included in these studies is small and no further conclusion can be drawn from these results.

Since platinum-drug resistance is thought to be multifactorial the involvement of other resistance mechanisms could have overruled the possible contribution of MMR status. However, platinum treatment does seem to select for MMR deficient cells since *in vitro *enrichment for MLH1 deficient colon cancer HCT116 cells in a mixed cell population was seen after cisplatin treatment [53]. In addition, several *in vivo *studies found an increase in the percentage of MSI and MLH1 methylation after platinum-based chemotherapy as well as a decrease in the percentage of cells positive for MLH1 and MSH2 [14,19,25,52]. Moreover, an increase in MLH1 methylation after platinum-based chemotherapy was associated with poor survival in ovarian cancer patients [19]. These results as well as the *in vitro *studies mentioned in the introduction, suggest that MMR inactivation causes a low level resistance to platinum-based chemotherapy which does not play a significant role in intrinsic resistance. However, due to selection during chemotherapy MMR inactivation might play a greater role in the acquired resistance. We therefore propose that the role of MMR inactivation in acquired resistance in ovarian cancer should be further investigated.

## Conclusion

No MMR inactivation was detected in 75 ovarian carcinoma specimens and no association was seen between MMR inactivation and resistance in the ovarian cancer cell lines as well as the ovarian carcinomas. We hypothesize that MMR inactivation is not clearly associated with intrinsic resistance in ovarian cancer. However, it might play a role in acquired resistance due to selection of MMR deficient cells during platinum-based chemotherapy, but this needs further investigation.

## Competing interests

The author(s) declare that they have no competing interests.

## Authors' contributions

JH contributed to the design of the study, did part of the methylation specific PCR, performed the statistical analysis and wrote the manuscript. IvS performed the quantitative RT-PCR and optimized the methylation specific PCR. WD supervised the microsatellite stability analysis and contributed to the manuscript. PvK carried out the methylation specific PCR. KR performed all quantitative RT-PCRs. PE reviewed the histopathology of the patients and assisted to the manuscript. MvdB provided all clinical follow-up and contributed to the manuscript. GS supervised the study. EB is the principle investigator who participated in the study-design, coordination, draft and finalization of the manuscript. All authors read and approved the final manuscript.

## Pre-publication history

The pre-publication history for this paper can be accessed here:



## Figures and Tables

**Table 1:** Patient and tumor characteristics.

Patient and tumor characteristics	No. of patients
FIGO stage	
Early (I-IIA)	20
Advanced (IIB-IV)	45
Unknown	10
Histological type	
Serous adenocarcinoma	36
Endometrioid adenocarcinoma	13
Mucinous adenocarcinoma	10
Clear cell adenocarcinoma	3
Mixed Mullerian Tumor	8
Poorly differentiated	3
Unknown	2
Tumor grade	
1	12
2	29
3	27
Unknown	7
Residual disease	
None	25
≤ 1 cm	15
> 1 cm	22
Unknown	13
Chemotherapy	
Cisplatin & endoxan	45 *
Carboplatin & endoxan	1 *
Other, not platinum containing	5
No chemotherapy	11
Unknown	12
* Response to platinum-based chemotherapy	
No response	11
Response	34
Unknown	1

Total	75

**Table 2:** Summary of the literature: Frequency of MSI in ovarian cancer. The total number of MSI, the number of MS markers used for the analysis and the number of MSI per histological subtype (if mentioned) is given for each study.

	MSI		MS markers		Histology					
			NCI-CP	total	SE	EN	CC	MU	MM	PD
Helleman et al.	0/75	(0%)	2	2	0/36 (0%)	0/13 (0%)	0/3 (0%)	0/10 (0%)	0/8 (0%)	0/3 (0%)
Mesquita et al.2005	0/34	(0%)	1	2	0/26 (0%)	-	0/8 (0%)	-	-	-
Gifford et al. 2004	2/138	(1%)	3	6	Not mentioned					
Catasus et al. 2004	5/54	(9%)	5	5	EN, CC and MM histology					
Cai et al.2004	6/42	(14%)	5	5	-	-	6/42 (14%)	-	-	-
Liu et al.2004	15/74	(20%)	4	4	-	15/74 (20%)	-	-	-	-
Singer et al.2004	6/75	(8%)	5	11	5/53 (9%)	1/14 (7%)	0/3 (0%)	0/5 (0%)	-	-
Geisler et al.2003	21/125	(17%)	5	6	10/69 (14%)	6/22 (27%)	0/4 (0%)	2/9 (22%)	-	1/2 (50%)
Watanabe et al. 2001	2/24	(8%)	5	10	Not mentioned					
Sood et al. 2001	13/109	(12%)	5	14	Not mentioned					
Gras et al.2001	7/76	(9%)	5	10	0/17 (0%)	1/48 (2%)	1/8 (13%)	0/2 (0%)	0/6 (0%)	-
Buller et al. 2001	22/56	(39%)	5	6	Not mentioned					
Chiaravalli et al.2001	3/16	(19%)	2	3	0/8 (0%)	1/2 (50%)	-	2/4 (50%)	-	0/2 (0%)
Ohwada et al.2000	15/61	(25%)	1	5	4/32 (13%)	-	-	11/29 (38%)	-	-
Allen et al.2000	1/25	(4%)	2	4	1/16 (6%)	0/2 (0%)	0/2 (0%)	0/1 (0%)	-	0/4 (0%)
Colella et al. 1998	3/20	(15%)	0	3	Not mentioned					
Sood et al. 1997	13/78	(17%)	1	9	Not mentioned					
Sood et al. 1996	11/68	(16%)	1	9	34 serous and 34 not serous					
Tangir et al.1996	2/31	(6%)	0	13	2/31 (6%)	-	-	-	-	-
Arzimanoglou et al. 1996	11/90	(12%)	1	3	Not mentioned					
Fujita et al.1995	7/44	(16%)	0	4	2/22 (9%)	5/10 (50%)	0/4 (0%)	0/8 (0%)	-	-

Total	165/1315	(13%)			24/310 (8%)	29/185 (16%)	7/74 (9%)	15/68 (22%)	0/14 (0%)	1/11 (9%)

Abbreviations: SE serous, EN endometrioid, CC clear cell, MU mucinous, MM mixed mullerian, PD poorly differentiated, - not present, MS microsatellite, NCI-CP National Cancer Institute Consensus Panel.

## References

[B1] Jemal A, Murray T, Samuels A, Ghafoor A, Ward E, Thun MJ (2003). Cancer statistics, 2003. CA Cancer J Clin.

[B2] van der Burg ME, van Lent M, Buyse M, Kobierska A, Colombo N, Favalli G, Lacave AJ, Nardi M, Renard J, Pecorelli S (1995). The effect of debulking surgery after induction chemotherapy on the prognosis in advanced epithelial ovarian cancer. Gynecological Cancer Cooperative Group of the European Organization for Research and Treatment of Cancer. N Engl J Med.

[B3] Cannistra SA (2004). Cancer of the ovary. N Engl J Med.

[B4] Hartmann LC, Lu KH, Linette GP, Cliby WA, Kalli KR, Gershenson D, Bast RC, Stec J, Iartchouk N, Smith DI, Ross JS, Hoersch S, Shridhar V, Lillie J, Kaufmann SH, Clark EA, Damokosh AI (2005). Gene expression profiles predict early relapse in ovarian cancer after platinum-paclitaxel chemotherapy. Clin Cancer Res.

[B5] Helleman J, Jansen MP, Span PN, van Staveren IL, Massuger LF, Meijer-van Gelder ME, Sweep FC, Ewing PC, van der Burg ME, Stoter G, Nooter K, Berns EM (2006). Molecular profiling of platinum resistant ovarian cancer. Int J Cancer.

[B6] Spentzos D, Levine DA, Kolia S, Otu H, Boyd J, Libermann TA, Cannistra SA (2005). Unique gene expression profile based on pathologic response in epithelial ovarian cancer. J Clin Oncol.

[B7] Maga G, Hubscher U (2003). Proliferating cell nuclear antigen (PCNA): a dancer with many partners. J Cell Sci.

[B8] Bellacosa A (2001). Functional interactions and signaling properties of mammalian DNA mismatch repair proteins. Cell Death Differ.

[B9] Peltomaki P (2003). Role of DNA mismatch repair defects in the pathogenesis of human cancer. J Clin Oncol.

[B10] Wei K, Kucherlapati R, Edelmann W (2002). Mouse models for human DNA mismatch-repair gene defects. Trends Mol Med.

[B11] Drummond JT, Anthoney A, Brown R, Modrich P (1996). Cisplatin and adriamycin resistance are associated with MutLalpha and mismatch repair deficiency in an ovarian tumor cell line. J Biol Chem.

[B12] Strathdee G, MacKean MJ, Illand M, Brown R (1999). A role for methylation of the hMLH1 promoter in loss of hMLH1 expression and drug resistance in ovarian cancer. Oncogene.

[B13] Lage H, Dietel M (1999). Involvement of the DNA mismatch repair system in antineoplastic drug resistance. J Cancer Res Clin Oncol.

[B14] Brown R, Hirst GL, Gallagher WM, McIlwrath AJ, Margison GP, van der Zee AG, Anthoney DA (1997). hMLH1 expression and cellular responses of ovarian tumour cells to treatment with cytotoxic anticancer agents. Oncogene.

[B15] Aebi S, Kurdi-Haidar B, Gordon R, Cenni B, Zheng H, Fink D, Christen RD, Boland CR, Koi M, Fishel R, Howell SB (1996). Loss of DNA mismatch repair in acquired resistance to cisplatin. Cancer Res.

[B16] Fink D, Nebel S, Aebi S, Zheng H, Cenni B, Nehme A, Christen RD, Howell SB (1996). The role of DNA mismatch repair in platinum drug resistance. Cancer Res.

[B17] de las Alas MM, Aebi S, Fink D, Howell SB, Los G (1997). Loss of DNA mismatch repair: effects on the rate of mutation to drug resistance. J Natl Cancer Inst.

[B18] Mesquita B, Veiga I, Pereira D, Tavares A, Pinto IM, Pinto C, Teixeira MR, Castedo S (2005). No significant role for beta tubulin mutations and mismatch repair defects in ovarian cancer resistance to paclitaxel/cisplatin. BMC Cancer.

[B19] Gifford G, Paul J, Vasey PA, Kaye SB, Brown R (2004). The acquisition of hMLH1 methylation in plasma DNA after chemotherapy predicts poor survival for ovarian cancer patients. Clin Cancer Res.

[B20] Catasus L, Bussaglia E, Rodrguez I, Gallardo A, Pons C, Irving JA, Prat J (2004). Molecular genetic alterations in endometrioid carcinomas of the ovary: similar frequency of beta-catenin abnormalities but lower rate of microsatellite instability and PTEN alterations than in uterine endometrioid carcinomas. Hum Pathol.

[B21] Cai KQ, Albarracin C, Rosen D, Zhong R, Zheng W, Luthra R, Broaddus R, Liu J (2004). Microsatellite instability and alteration of the expression of hMLH1 and hMSH2 in ovarian clear cell carcinoma. Hum Pathol.

[B22] Liu J, Albarracin CT, Chang KH, Thompson-Lanza JA, Zheng W, Gershenson DM, Broaddus R, Luthra R (2004). Microsatellite instability and expression of hMLH1 and hMSH2 proteins in ovarian endometrioid cancer. Mod Pathol.

[B23] Singer G, Kallinowski T, Hartmann A, Dietmaier W, Wild PJ, Schraml P, Sauter G, Mihatsch MJ, Moch H (2004). Different types of microsatellite instability in ovarian carcinoma. Int J Cancer.

[B24] Geisler JP, Goodheart MJ, Sood AK, Holmes RJ, Hatterman-Zogg MA, Buller RE (2003). Mismatch repair gene expression defects contribute to microsatellite instability in ovarian carcinoma. Cancer.

[B25] Watanabe Y, Koi M, Hemmi H, Hoshai H, Noda K (2001). A change in microsatellite instability caused by cisplatin-based chemotherapy of ovarian cancer. Br J Cancer.

[B26] Sood AK, Holmes R, Hendrix MJ, Buller RE (2001). Application of the National Cancer Institute international criteria for determination of microsatellite instability in ovarian cancer. Cancer Res.

[B27] Gras E, Catasus L, Arguelles R, Moreno-Bueno G, Palacios J, Gamallo C, Matias-Guiu X, Prat J (2001). Microsatellite instability, MLH-1 promoter hypermethylation, and frameshift mutations at coding mononucleotide repeat microsatellites in ovarian tumors. Cancer.

[B28] Buller RE, Shahin MS, Holmes RW, Hatterman M, Kirby PA, Sood AK (2001). p53 Mutations and microsatellite instability in ovarian cancer: Yin and yang. Am J Obstet Gynecol.

[B29] Chiaravalli AM, Furlan D, Facco C, Tibiletti MG, Dionigi A, Casati B, Albarello L, Riva C, Capella C (2001). Immunohistochemical pattern of hMSH2/hMLH1 in familial and sporadic colorectal, gastric, endometrial and ovarian carcinomas with instability in microsatellite sequences. Virchows Arch.

[B30] Ohwada M, Suzuki M, Saga Y, Sato I (2000). DNA replication errors are frequent in mucinous cystadenocarcinoma of the ovary. Cancer Genet Cytogenet.

[B31] Allen HJ, DiCioccio RA, Hohmann P, Piver MS, Tworek H (2000). Microsatellite instability in ovarian and other pelvic carcinomas. Cancer Genet Cytogenet.

[B32] Colella G, Vikhanskaya F, Codegoni AM, Bonazzi C, D'Incalci M, Broggini M (1998). hMLH1 and hMSH2 expression and BAX frameshift mutations in ovarian cancer cell lines and tumors. Carcinogenesis.

[B33] Sood AK, Skilling JS, Buller RE (1997). Ovarian cancer genomic instability correlates with p53 frameshift mutations. Cancer Res.

[B34] Sood AK, Buller RE (1996). Genomic instability in ovarian cancer: a reassessment using an arbitrarily primed polymerase chain reaction. Oncogene.

[B35] Tangir J, Loughridge NS, Berkowitz RS, Muto MG, Bell DA, Welch WR, Mok SC (1996). Frequent microsatellite instability in epithelial borderline ovarian tumors. Cancer Res.

[B36] Arzimanoglou II, Lallas T, Osborne M, Barber H, Gilbert F (1996). Microsatellite instability differences between familial and sporadic ovarian cancers. Carcinogenesis.

[B37] Fujita M, Enomoto T, Yoshino K, Nomura T, Buzard GS, Inoue M, Okudaira Y (1995). Microsatellite instability and alterations in the hMSH2 gene in human ovarian cancer. Int J Cancer.

[B38] Burger H, Nooter K, Boersma AW, Kortland CJ, Stoter G (1997). Lack of correlation between cisplatin-induced apoptosis, p53 status and expression of Bcl-2 family proteins in testicular germ cell tumour cell lines. Int J Cancer.

[B39] Schuyer M, van der Burg ME, Henzen-Logmans SC, Fieret JH, Klijn JG, Look MP, Foekens JA, Stoter G, Berns EM (2001). Reduced expression of BAX is associated with poor prognosis in patients with epithelial ovarian cancer: a multifactorial analysis of TP53, p21, BAX and BCL-2. Br J Cancer.

[B40] Westenend PJ, Schutte R, Hoogmans MM, Wagner A, Dinjens WN (2005). Breast cancer in an MSH2 gene mutation carrier. Hum Pathol.

[B41] Arita M, Zhong X, Min Z, Hemmi H, Shimatake H (2003). Multiple sites required for expression in 5'-flanking region of the hMLH1 gene. Gene.

[B42] Deng G, Chen A, Pong E, Kim YS (2001). Methylation in hMLH1 promoter interferes with its binding to transcription factor CBF and inhibits gene expression. Oncogene.

[B43] Herman JG, Graff JR, Myohanen S, Nelkin BD, Baylin SB (1996). Methylation-specific PCR: a novel PCR assay for methylation status of CpG islands. Proc Natl Acad Sci U S A.

[B44] Taverna P, Liu L, Hanson AJ, Monks A, Gerson SL (2000). Characterization of MLH1 and MSH2 DNA mismatch repair proteins in cell lines of the NCI anticancer drug screen. Cancer Chemother Pharmacol.

[B45] Carethers JM, Pham TT (2000). Mutations of transforming growth factor beta 1 type II receptor, BAX, and insulin-like growth factor II receptor genes in microsatellite unstable cell lines. In Vivo.

[B46] Branch P, Masson M, Aquilina G, Bignami M, Karran P (2000). Spontaneous development of drug resistance: mismatch repair and p53 defects in resistance to cisplatin in human tumor cells. Oncogene.

[B47] Aquilina G, Ceccotti S, Martinelli S, Soddu S, Crescenzi M, Branch P, Karran P, Bignami M (2000). Mismatch repair and p53 independently affect sensitivity to N-(2-chloroethyl)-N'-cyclohexyl-N-nitrosourea. Clin Cancer Res.

[B48] Boland CR, Thibodeau SN, Hamilton SR, Sidransky D, Eshleman JR, Burt RW, Meltzer SJ, Rodriguez-Bigas MA, Fodde R, Ranzani GN, Srivastava S (1998). A National Cancer Institute Workshop on Microsatellite Instability for cancer detection and familial predisposition: development of international criteria for the determination of microsatellite instability in colorectal cancer. Cancer Res.

[B49] Catasus L, Machin P, Matias-Guiu X, Prat J (1998). Microsatellite instability in endometrial carcinomas: clinicopathologic correlations in a series of 42 cases. Hum Pathol.

[B50] Caduff RF, Johnston CM, Svoboda-Newman SM, Poy EL, Merajver SD, Frank TS (1996). Clinical and pathological significance of microsatellite instability in sporadic endometrial carcinoma. Am J Pathol.

[B51] Risinger JI, Berchuck A, Kohler MF, Watson P, Lynch HT, Boyd J (1993). Genetic instability of microsatellites in endometrial carcinoma. Cancer Res.

[B52] Samimi G, Fink D, Varki NM, Husain A, Hoskins WJ, Alberts DS, Howell SB (2000). Analysis of MLH1 and MSH2 expression in ovarian cancer before and after platinum drug-based chemotherapy. Clin Cancer Res.

[B53] Fink D, Zheng H, Nebel S, Norris PS, Aebi S, Lin TP, Nehme A, Christen RD, Haas M, MacLeod CL, Howell SB (1997). In vitro and in vivo resistance to cisplatin in cells that have lost DNA mismatch repair. Cancer Res.

